# Association of Glycosylated Hemoglobin with Long-Term Adverse Cardiac Events after Percutaneous Coronary Intervention in Non Diabetes and Controlled Diabetes Patients: An Observational Study from the Korean COACT Registry

**DOI:** 10.3390/life12111945

**Published:** 2022-11-21

**Authors:** Ha-Wook Park, Sung-Ho Her, Jin Jung, Hyunji Chun, Wook-Sung Chung

**Affiliations:** 1 Division of Cardiology Cardiovascular Center, Bucheon Sejong Hospital, Bucheon 14754, Republic of Korea; 2 Department of Cardiology, St. Vincent’s Hospital, College of Medicine, The Catholic University of Korea, Seoul 16247, Republic of Korea; 3 Division of Cardiology, College of Medicine, The Catholic University of Korea, Seoul 06591, Republic of Korea

**Keywords:** glycosylated hemoglobin, percutaneous coronary intervention, diabetes mellitus, adverse cardiac event

## Abstract

Glycosylated hemoglobin (HbA1c) is an established marker associated with cardiovascular risk, even if it is below the diagnostic threshold for diabetes mellitus (DM). However, whether or not prediabetic and controlled diabetic levels of HbA1c are associated with increased major adverse cardiovascular events (MACE) after percutaneous coronary intervention (PCI) remains unclear. This observational study included a total of 9128 patients who underwent PCI in the COACT registry from eight centers in Korea. A total of 2517 non-DM patients were divided into three groups (Groups I, II, III) according to their HbA1c levels and compared with 965 controlled DM patients (HbA1c < 7.0%, Group IV). During 22 months of median follow-up, there was no significant differences in MACE (*p* = 0.294) and cardiac death (*p* = 0.105) among the four groups. In addition, there were also no significant differences in MACE (*p* = 0.058) between Group III and Group IV. Although patients were diagnosed as DM, they had a similar prognosis in the same range of newly diagnosed DM patients in HbA1c, if they were treated well. The results of this study suggest that intensive treatment is required to reach the Hba1c target in diabetic patients with PCI in order to have a similar prognosis to patients not previously diagnosed with diabetes.

## 1. Introduction

Numerous studies have reported the relationship between abnormal glucose tolerance and future cardiovascular events. Bartnik et al. [1] reported that abnormal glucose tolerance (newly diagnosed type 2 diabetes mellitus (DM) or impaired glucose tolerance) is a strong risk factor for future cardiovascular events after myocardial infarction. Recently, glycosylated hemoglobin (HbA1c) is regarded as a superior predictor than fasting glucose for assessing the long-term risk of subsequent cardiovascular disease in non-diabetic patients [2]. However, whether non-diabetic levels of HbA1c are associated with major adverse cardiovascular events (MACE) after percutaneous coronary intervention (PCI) remains unclear. Thus, the objective of this study was to investigate the relationship of HbA1c levels with long-term risk of MACE using the observational registry data of non-diabetic and controlled diabetic patients with PCI.

## 2. Materials and Methods

### 2.1. Study Design and Population

**Figure 1:**
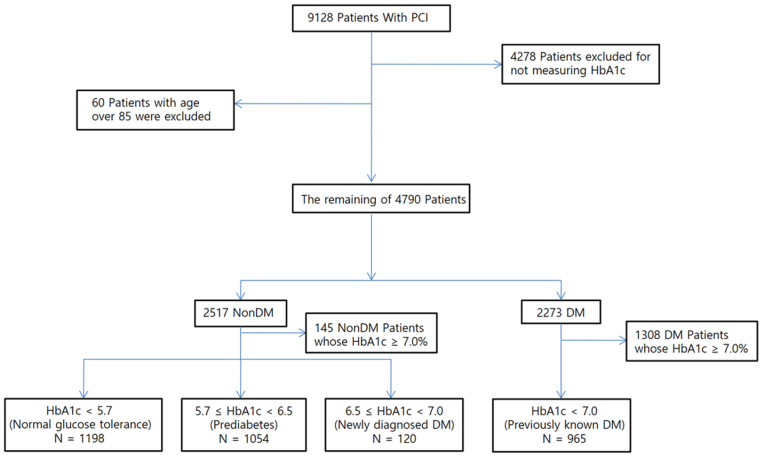
Formation of study population. PCI, percutaneous coronary intervention; HbA1c, glycosylated hemoglobin; DM, diabetes mellitus.

COACT (Catholic University of Korea: percutaneous coronary intervention) is a large, retrospective observational registry of demographic, clinical, and procedural data, including short-term and long-term clinical outcomes of all patients undergoing PCI with the use of DES from eight affiliated hospitals of the Catholic University of Korea between January 2004 and December 2009. These hospitals are located throughout the country, and all provide high-volume PCI (more than 500 cases per year). There was no industry involvement in the design, conduct, or analysis of the study. The study protocol was approved by the Institutional Review Board (IRB) of each participating institution. The inclusion period was between January 2004 and December 2009. A total of 9218 patients were registered in COACT. Figure 1 shows a flow diagram for the selection of the study population. In the present study, among a total of 9128 registered patients who had undergone successful PCI, 3337 patients were enrolled according to the following including criteria: (1) those with HbA1c measured at admission; (2) those aged less than 85 years. Patients who had no previous history of DM (non-DM and newly diagnosed DM) and patients with known DM were classified into one of the following categories according to HbA1 levels.

The study flow chart is summarized in Figure 1. We selected 4790 patients, who had HbA1c measured on admission and were under 85 years old. Of these, 2517 patients were not diagnosed as DM before hospitalization and 2273 patients had DM on admission. Patients with HbA1c > 7.0% was excluded. Final analysis was therefore performed on 3337 patients.

Patients were divided on the basis of admission HbA1c level in non-DM patients: Group I, HbA1c < 5.7% (*n* = 1198); Group II, 5.7% ≤ HbA1c < 6.5% (*n* = 1054); Group III (new-diagnosed DM), HbA1c ≥ 6.5% (*n* = 120). We also assembled another group of DM patients, which were relatively well controlled (Group IV, HbA1c < 7.0%, (*n* = 965)).

### 2.2. Measurement of HbA1c

The measurement of HbA1c was accomplished by assay of whole-blood samples drawn at admission using high-performance liquid chromatography. The reference method for HbA1c was performed utilizing a Roche Modular P800 analyzer (Roche Diagnostics Basel, Switzerland). All instruments used were standardized to the Diabetes Control and Complications Trial assay.

### 2.3. PCI

Conventional coronary angiography was performed in a standard manner. All patients were administered loading doses of aspirin (300 mg) and clopidogrel (300–600 mg) unless contraindicated. PCI was performed using a standard technique. The selection of stenting techniques and stent types was left to the operator’s discretion. Before PCI, all patients received aspirin 300 mg daily. Clopidogrel (600 mg loading dose) was given at least 1 day before the procedure. The procedure was performed after administration of unfractionated heparin (100 U/kg). The choice of stent was at each physician’s discretion. The stent was deployed after balloon angioplasty. A successful PCI procedure was defined as a decrease in a minimum stenosis diameter to <30%, with thrombolysis in myocardial infarction grade III flow on coronary angiogram. After discharge, patients continued receiving the same medications, including dual antiplatelet medication. After the procedure, aspirin treatment (100–200 mg once daily) was continued throughout the study period.

### 2.4. Data Collection

Demographic, clinical, laboratory, angiographic, and outcome data were collected using an internet-based reporting system. All baseline and procedural coronary angiograms were digitally stored on either a compact disc or a hard disk in digital imaging and communication in Medicine format. A total of 9128 patients who underwent PCI for coronary artery disease were enrolled in COACT.

### 2.5. Study Endpoint and Definitions

Clinical events were defined based on the recommendations of the Academic Research Consortium (ARC) [3]. Any death due to proximate cardiac cause (e.g., MI, low-output failure, fatal arrhythmia), unwitnessed death, death of unknown cause, and all procedure-related deaths, including those related to concomitant treatment, were classified as cardiac death. History of smoking was a composite of current smoker and ex-smoker. Individuals with a fasting blood glucose ≥ 126 mg/dL or clinical symptoms and random serum glucose > 200 mg/dl were diagnosed as DM, which was treated with diet and oral glucose-lowering medication. Diagnosis of hypertension (in untreated subjects) was based on accepted criteria—over the mean of 140 mmHg for systolic blood pressure (SBP) and/or 90mmHg for diastolic blood pressure (DBP). MI was defined as the presence of clinical signs of MI combined with a creatine kinase MB fraction (CK-MB) or troponin-T/troponin-I increase more than the upper normal limit that was not related to an interventional procedure. Target lesion revascularization (TLR) was defined as a repeat PCI of the lesion within 5 mm of deployed stent or bypass graft surgery of the target vessel. All patients underwent transthoracic echocardiography (TTE) with standard acoustic windows. Ejection fraction was measured by TTE.

### 2.6. Statistical Analysis

SPSS software version 18.0 (IBM SPSS, Chicago, IL, USA) was used for all statistical analyses. All continuous variables are reported as mean ± standard deviation (SD). Stu-dent’s *t*-test was used for continuous variables. A chi-square test was used for categorical data. Event-free survival curves were constructed using Kaplan–Meier estimates. They were compared with the log-rank test. Logistic regression analysis was used to identify the relationship between HbA1c and MACE. We also used a Cox proportional hazards model to estimate multivariate-adjusted odds ratios (ORs) for MACE and subtypes of adverse cardiovascular events according to HbA1c values. Statistical significance was accepted when a two-sided *p*-value was less than 0.05.

## 3. Results

Baseline and angiographic characteristics are shown in Table 1. For the total group, the median follow-up period was 22 months. In non-DM and newly diagnosed DM patients, those with higher HbA1c levels were older and more often females with higher LDL and triglyceride. In addition, they more often had a prior history of coronary artery disease. However, there was no significant difference in each group for patients who were diagnosed as MI at admission.

**Figure 2:**
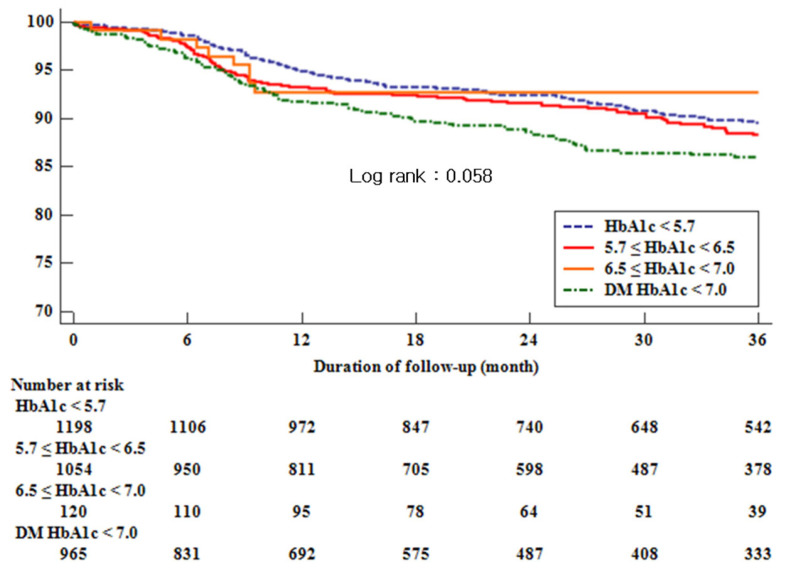
Kaplan–Meier curves showing event-free survival from major cardiac adverse event (MACE) after percutaneous coronary intervention (PCI) during the 4-year follow-up. HbA1c, glycosylated hemoglobin.

We compared Group IV (DM) with Group III (new-diagnosed DM). Group IV showed higher serum creatinine levels but lower levels of hemoglobin, total cholesterol, and tri-glyceride LDL. In the DM group, 79% of patients were treated with an oral agent for glucose control. Table 2 (by Cox regression analysis) also displays a comparison of each group in MACE (Group I vs. Group II vs. Group III vs. Group IV; 9.9% vs. 10.0% vs. 8.3% vs. 12.0%, *p* = 0.294) and cardiac death (1.3% vs. 1.2% vs. 0.8% vs. 2.4%, *p* = 0.105). Non-diabetic HbA1c and HbA1c < 7.0% level showed no definite correlations with MACE and cardiac death. However, comparison of groups showed significant difference only in all deaths that were not directly related to cardiovascular events (5.5% vs. 5.9% vs. 9.2% vs. 12.0%, *p* < 0.001). Long-term clinical outcome (survival free from MACE) is displayed in Figure 2. Non-diabetic HbA1c and HbA1c < 7.0% level was not statistically significant, showing similar poor prognosis in every group.

We calculated unadjusted and multivariate adjusted odds ratio (OR) for MACE according to four groups. We compared different groups with Group I. Multivariate adjusted odds ratio (with 95% confidence intervals; CI) for MACE was 0.92 (95%CI: 0.66–1.28, *p* = 0.612) for Group II, 0.67 (95%CI: 0.31–1.45, *p* = 0.313) for Group III, and 0.91 (95%CI: 0.62–1.34, *p* = 0.630) for Group IV. Multivariate adjusted odds ratio for cardiac death was 1.34 (95%CI: 0.57–3.16, *p* = 0.501) for Group II, 1.29 (95%CI: 0.14–11.65, *p* = 0.824) for Group III, and 1.69 (95%CI: 0.72–3.94, *p* = 0.226) for Group IV (Table 3).

## 4. Discussion

This paper compared MACEs in non-DM, newly diagnosed DM, and known DM patients treated with PCI. HbA1c has several advantages as a diagnostic test: it has higher repeatability and can be assessed in the non-fasting state [2]. Although prior epidemiological studies have shown that increasing levels of HbA1c are associated with an increased incidence of macrovascular disease including CVD [4,5,6,7,8], this study provides evidence that HbA1c level was not an important predictive factor in patients with non-diabetic HbA1c and HbA1c < 7.0% level.

It is a remarkable point that HbA1c is not a powerful predictor in non-DM patients. Non-diabetic level of HbA1c level does not show MACE differences in our data. In our study, the incidence of stroke was higher in prediabetes patients than in patients with normal blood glucose levels. The prevalence of prediabetes in previously normoglycemic nondiabetic patients who had suffered a recent TIA or an ischemic stroke was appoximately 37%, ranging from 31% to 53% within the first 90 days after the vascular event (acute phase) and approximately 32% in the post-acute phase (ranging from 23% to 46%) [4]. The majority of acute stroke patients had disorders of glucose metabolism. In most cases, this fact has been unrecognized. DM and prediabetes can worsen acute stroke outcomes, and therefore effect the post-acute/chronic phase [4].

Next, well-controlled DM patients with PCI (Group IV) and newly diagnosed DM patients who were in same range of HbA1c had no significant difference. DM is a major risk factor for coronary heart disease (CHD) mortality. It is also associated with decreased survival after MI [5]. However, prognosis does not depend on whether a patient is classified as DM or not anymore. Several studies have proven that HbA1c has a predictive value for CVD complications in a population (DM or non-DM) [2,6,7,8,9,10,11]. Well-controlled HbA1c reveals that patients get well treatment. As for the target of DM treatment, ADA recommends that HbA1c level should be lower than 7.0% [12]. In our study, the incidence of MACE and cardiac death was similar in groups with HbA1c < 7.0%, whether patients had DM or not who received PCI. There was a difference in all death, especially in Groups 3 and 4, which seems to be due to diabetes not only affecting cardiovascular but also to other target organ damage.

As shown in this study, Group IV (well controlled DM group) had lower levels of cholesterol than Group III (newly diagnosed DM). This might be caused by administration of statin, a lipid lowering agent. After they were diagnosed as DM, as recommended by guidelines [12], they were checked for lipid profile and administrated with statin at a higher level than those in the non-DM group (Table 1).

Risk factors such as old age, CVA history, hypertension, and so on, were more increased in Group IV compared with Group III. The use of antithrombotic drugs such as aspirin would be more frequent (Table 1). Thus, the hemoglobin levels of Group IV were lower than those of Group III, probably due to old age, chronic renal failure, and the use of aspirin, etc.

## 5. Study Limitation

This study has several limitations as a nonrandomized, observational study. First, the range of non-DM patients; Group III (newly diagnosed DM) included HbA1c ≥ 6.5% (6.5 ≤ HbA1c < 7.0%), which is over the current DM criteria. In addition, our DM patients were diagnosed only based on fasting blood glucose and random glucose levels, not HbA1c levels, unlike current criteria. This was the reason why patients with HbA1c > 6.5% were categorized as newly diagnosed DM in this study. Second, there were no data on the duration of diabetes, the start of glucose-lowering medication, or the kind of treatment (per oral agent or insulin) during the follow-up period. Moreover, during the follow-up period, in this study, the alteration of HbA1c level from admission was unknown. Third, patients received PCI for various reasons, ranging from MI to stable angina. However, there was no significant difference. They already had target organ damage. In addition, compared to the general population, there were relatively more lighter-weight patients and fewer smokers. Thus, this population did not represent all DM patients.

## 6. Conclusions

In patients who had undergone PCI, non-diabetic HbA1c and HbA1c < 7.0% level were not associated with poor clinical outcomes in long-term follow-up. Remarkably, although patients were diagnosed as DM, they showed similar prognosis to newly diagnosed DM patients in HbA1c if they were treated well. Thus, the results of this study suggest that intensive treatment is required to reach the Hba1c target in diabetic patients with PCI in order to have a similar prognosis to patients not previously diagnosed with diabetes.

## Figures and Tables

**Table 1:** Baseline characteristics of study patients.

Variables	Non DM			Known DM	*p*-Value
	HbA1c < 5.7 Group I (N = 1198 )	5.7 ≤ HbA1c < 6.5 II (N = 1054 )	6.5 ≤ HbA1c < 7.0 III (N = 120)	HbA1c < 7.0 IV (N = 965)	
Male, n	863 (72.0)	701 (66.5)	70 (58.3)	403 (64.3)	<0.001
Age, year	60.6 ± 11.3	63.3 ± 10.1	64.8 ± 8.9	65.2 ± 9.6	<0.001
Body-mass index, kg/m2	24.5 ± 2.9	24.8 ± 3.1	25.1 ± 3.4	24.7 ± 3.1	0.054
Family History of CAD, n	70 (5.8)	55 (5.2)	3 (2.5)	51 (5.3)	0.469
History of smoking, n					
Never smoker	728 (60.8)	620 (58.8)	73 (60.8)	596 (61.8)	0.566
Ex-smoker/Current smoker	470 (39.2)	434 (41.2)	47 (39.2)	369 (38.2)	
Clinical History, n					
Hypertension, n	625 (52.2)	601 (57.0)	62 (51.7)	681 (70.6)	<0.001
Cerebrovascular disease, n	59 (4.9)	74 (7.0)	11 (9.2)	100 (10.4)	<0.001
Chronic renal failure, n	36 (3.0)	34 (3.2)	5 (4.2)	111 (11.5)	<0.001
Previous procedures(PTCA/CABG), n	95 (7.9)	110 (10.4)	11 (9.2)	85 (8.8)	0.224
FBS, mg/dL	108.0 ± 25.2	115.0 ± 29.8	129.2 ± 39.8	137.2 ± 51.1	<0.001
HbA1c, %	5.3 ± 0.2	6.0 ± 0.2	6.7 ± 0.1	6.2 ± 0.5	<0.001
Total cholesterol, mg/dL	177.8 ± 38.1	180.1 ± 38.9	179.1 ± 43.1	167.4 ± 38.4	<0.001
HDL –cholesterol, mg/dL	43.6 ± 10.9	42.3 ± 10.4	40.9 ± 9.4	40.8 ± 11.0	<0.001
Triglyceride, mg/dL	129.8 ± 83.7	138.1 ± 91.2	163.8 ± 128.9	142.1 ± 97.8	<0.001
LDL-cholesterol, mg/dL	108.2 ± 34.6	110.2 ± 35.0	106.3 ± 40.4	98.1 ± 33.5	<0.001
Serum creatinine, mg/dL	1.1 ± 1.0	1.1 ± 0.8	1.1 ± 0.8	1.4 ± 1.5	<0.001
Hb	13.5 ± 1.8	13.4 ± 1.8	13.2 ± 1.7	12.6 ± 2.0	<0.001
hsCRP	0.9 ± 2.2	1.1 ± 2.6	0.8 ± 1.5	1.6 ± 3.6	<0.001
Multi-vessel disease, n	560 (46.7)	599 (56.8)	68 (56.7)	590 (61.1)	<0.001
LVEF, %	58.9 ± 9.8	58.9 ± 10.5	57.7 ± 11.2	57.8 ± 11.8	0.062
Aspirin, n	1194 (98.8)	1038 (98.5)	116 (96.7)	931 (96.5)	<0.001
Statin, n	1043 (87.1)	905 (85.9)	103 (85.8)	774 (80.2)	<0.001
DM treatment					
None	1198 (100.0)	1054 (100.0)	120 (100.0)	0(0.0)	-
No treatment	0 (0.0)	0 (0.0)	0 (0.0)	771 (79.9)	
Oral hypoglycemic agents, n	0 (0.0)	0 (0.0)	0 (0.0)	95(9.8)	
Insulin therapy, n	0 (0.0)	0 (0.0)	0 (0.0)	99 (10.3)	

Data are expressed as mean (standard deviation) or number (%); DM, diabetes mellitus; HbA1c, glycosylated hemoglobin; CAD, coronary artery disease; PTCA, percutaneous transluminal coronary angioplasty; CABG, coronary artery bypass graft; FBS, fasting blood sugar; LVEF, left ventricular ejection fraction.

**Table 2:** Adverse cardiovascular events.

	Non DM			Known DM	*p*-Value
	HbA1c < 5.7 Group I (N = 1198)	5.7 ≤ HbA1c < 6.5 II (N = 1054)	6.5 ≤ HbA1c < 7.0 III (N = 120)	HbA1c < 7.0 IV (N = 965)	
**All death, *n* (%)**	66 (5.5)	62 (5.9)	11 (9.2)	116 (12.0)	<0.001
**Cardiac death, *n* (%)**	15 (1.3)	13 (1.2)	1 (0.8)	23 (2.4)	0.105
**Non fatal MI, *n* (%)**	12 (1.0)	8 (0.8)	0 (0.0)	10 (1.0)	0.645
**Stroke, *n* (%)**	9 (0.8)	20 (1.9)	0 (0.0)	29 (3.0)	<0.001
**TLR, *n* (%)**	93 (7.8)	72 (6.8)	8 (6.7)	65 (6.7)	0.769
**Total MACE (Cardiac Death/MI/Stroke/TLR), *n* (%)**	119 (9.9)	105 (10.0)	10 (8.3)	65 (12.0)	0.294

Data are expressed as mean (standard deviation) or number (%); DM, diabetes mellitus; HbA1c, glycosylated hemoglobin; MI, myocardial infarction; TLR, target lesion revascularization; MACE, major adverse cardiac event.

**Table 3:** Multivariate odds ratios for Major cardiac adverse event (MACE) and each clinical events by glycosylated hemoglobin (HbA1C) levels.

	Unadjusted			Multivariate Adjusted		
	ORs	95% CIs	*p*-Value	ORs	95% CIs	*p*-Value
**Cardiac death**						
HbA1c < 5.7	1	(reference)		1	(reference)	
5.7 ≤ HbA1c < 6.5	1.06	0.51–2.24	0.869	1.34	0.57–3.16	0.501
6.5 ≤ HbA1c < 7.0	0.74	0.10–5.63	0.775	1.29	0.14–11.65	0.824
DM HbA1c < 7.0	2.20	1.15–4.21	0.018	1.69	0.72–3.94	0.226
**Recurrent Nonfatal MI**						
HbA1c < 5.7	1	(reference)		1	(reference)	
5.7 ≤ HbA1c < 6.5	0.83	0.34–2.03	0.679	0.27	0.08–0.93	0.037
6.5 ≤ HbA1c < 7.0	–	–	–	–	–	–
DM HbA1c < 7.0	1.21	0.52–2.79	0.663	0.19	0.04–1.01	0.051
**Stroke**						
HbA1c < 5.7	1	(reference)		1	(reference)	
5.7 ≤ HbA1c < 6.5	2.72	1.24–5.99	0.013	2.41	0.99–5.87	0.053
6.5 ≤ HbA1c < 7.0	–	–	–	–	–	–
DM HbA1c < 7.0	4.60	2.18-9.73	<0.001	3.41	1.30–8.90	0.012
**TLR**						
HbA1c < 5.7	1	(reference)		1	(reference)	
5.7 ≤ HbA1c < 6.5	0.95	0.70–1.29	0.720	0.71	0.47–1.06	0.090
6.5 ≤ HbA1c < 7.0	0.95	0.46–1.96	0.891	0.51	0.21–1.27	0.149
DM HbA1c < 7.0	0.99	0.72–1.36	0.966	0.57	0.34–0.95	0.033
**Total MACE (Cardiac Death/MI/Stroke/TLR)**						
HbA1c < 5.7	1	(reference)		1	(reference)	
5.7 ≤ HbA1c < 6.5	1.08	0.83–1.41	0.559	0.92	0.66–1.28	0.612
6.5 ≤ HbA1c < 7.0	0.92	0.48–1.76	0.802	0.67	0.31–1.45	0.313
DM HbA1c < 7.0	1.39	1.08–1.80	0.001	0.91	0.62–1.34	0.630

Adjusted by sex, age, hypertension, cerebrovascular disease, chronic renal failure, total cholesterol, HDL cholesterol, Triglyceride, LDL cholesterol, serum creatinine, Hb, hsCRP, and multi-vessel disease. OR, odds ratio; CI, confidence interval; DM, diabetes mellitus; HbA1c, glycosylated hemoglobin; MI. myocardial infarction; TLR, target lesion revascularization.

## Data Availability

Not applicable.
